# 

**Published:** 1885-02

**Authors:** 


### CONTENTS.

COMMUNICATIONS.

Muriate of Cocaine ........ 57
Salivary Calculus ........ 59
Microscopical Studies Upon the Absorption of the Roots of Temporary Teeth ........ 64

SELECTIONS.

On Lancing the Gums ........ 69
Gold Leaf ........ 73
On Assuring Healthy Dentine Over Endangered Pulps ........ 75
A Gas Furnace for Firing Dental Enamels and Porcelains, Causes of "Gasing" in Firing Teeth ........ 79
Notes on a case of Poisoning from Mrs. Winslow's Soothing Syrup... 81
Civilization and the Teeth ........ 83
General Precautions in the Administration of Anæsthetics ........ 85
The Ideal Physician ........ 87
The Human Face ........ 88
Steel Uniformity ........ 90
New Dental Institute in Berlin ........ 92
The Cheapest Antiseptic ........ 92
The Precipitation of Gold ........ 93
Idunium ........ 93
A Preparation of Magnesium ........ 94
A Beautiful Slide ........ 94
Engine Brush ........ 94
Metal Therapeutics ........ 95
Disinfection by the Fumes of Sulphide of Carbon ........ 95
Affections of the Gum in Relation to other Diseases ........ 96
Regulations Governing the Height of Houses ........ 96
Water and Constipation ........ 96
SALMAGUNDI ........ 97

EDITORIAL.

Transfer of Neuralgia ........ 104
Meeting ........ 106
Chronic Nasal Catarrh ........ 107
College Association ........ 107
Alumni Meeting ........ 107
Commencement ........ 108
Married ........ 108

